# The Role of Cognitive Factors in Childhood Social Anxiety: Social Threat Thoughts and Social Skills Perception

**DOI:** 10.1007/s10608-016-9821-x

**Published:** 2016-11-17

**Authors:** Rianne E. van Niekerk, Anke M. Klein, Esther Allart-van Dam, Jennifer L. Hudson, Mike Rinck, Giel J. M. Hutschemaekers, Eni S. Becker

**Affiliations:** 1 Behavioural Science Institute, Radboud University Nijmegen, P.O. Box 9104, 6500 HE Nijmegen, The Netherlands; 2 Pro Persona, Centre for Anxiety Disorders Overwaal, Nijmegen, The Netherlands; 3 Centre for Emotional Health, Macquarie University, Sydney, Australia

**Keywords:** Social anxiety, Social threat thoughts, Social skills, Speech task, Children

## Abstract

Models of cognitive processing in anxiety disorders state that socially anxious children display several distorted cognitive processes that maintain their anxiety. The present study investigated the role of social threat thoughts and social skills perception in relation to childhood trait and state social anxiety. In total, 141 children varying in their levels of social anxiety performed a short speech task in front of a camera and filled out self-reports about their trait social anxiety, state anxiety, social skills perception and social threat thoughts. Results showed that social threat thoughts mediated the relationship between trait social anxiety and state anxiety after the speech task, even when controlling for baseline state anxiety. Furthermore, we found that children with higher trait anxiety and more social threat thoughts had a lower perception of their social skills, but did not display a social skills deficit. These results provide evidence for the applicability of the cognitive social anxiety model to children.

## Introduction

Fear in social situations can be very impairing: Having to give a presentation at school, or being the centre of attention is a frightening experience for many children. A persistent strong fear of these types of situations has been recognized by 22% of the boys and 32% of the girls between the age of 14–24 (Wittchen et al. 1999). When social anxiety generalizes, it has a significant impact on children’s functioning, influencing self-esteem, schooling, peer relationships, and their family environment (Essau et al. 2000; Ginsburg et al. 1998). In addition, anxiety that starts in childhood often persists into adulthood when left untreated (Kessler et al. 2005). Considering the grave impact on many life areas and the long lasting effects of social anxiety, research about factors that underlie and maintain social anxiety in children is essential.

Why do children become frightened in specific social situations like giving a presentation? It is to be expected that children who have a more anxious disposition are the ones who become more anxious in these situations, because theoretically “trait anxiety predicts state anxiety under conditions of psychological threat, especially conditions of evaluation” (p. 209, Reiss 1997). However, several studies showed that trait social anxiety alone only moderately predicts elevated anxiety during a speech. It is of interest to find more factors that may relate to state anxiety, for this will offer more insights into the mechanisms by which anxiety arises. These insights can be used for improving treatment interventions.

Based on cognitive theories of the development of social anxiety (Clark and Wells 1995; Hofmann 2007; Rapee and Heimberg 1997), social threat cognitions might be important for predicting which children will become anxious during a social evaluative task such as giving a speech (Hodson et al. 2008). According to the theory by Rapee and Heimberg (1997), socially anxious individuals are greatly concerned that they will be negatively evaluated and see others as inherently critical. They also tend to perceive themselves as less socially skilful (Alfano et al. 2006; Foa et al. 1996; Hofmann 2004). Despite regular exposure to social situations, anxiety does not decrease, because socially anxious individuals are convinced that their negative beliefs are being confirmed (Clark 2001). The expectancy of social threat, for example expecting to be bullied or to be rejected (Hofmann 2007) and the anticipation of social mishaps is seen as a maintaining factor in social anxiety, because socially anxious individuals fear that the next social event will have a negative outcome as well. As a result, socially anxious individuals engage in avoidance and safety behaviour, preventing opportunities in which the negative beliefs can be disconfirmed (Wells et al. 1995).

The link between cognitive factors and state anxiety in children has been investigated by Tuschen-Caffier et al. (2011), who focussed on negative self-evaluation in children with various levels of social anxiety. They found a relationship between trait anxiety, state anxiety and negative thoughts: the higher the trait anxiety, the higher the state anxiety during a speech task, and the more negative evaluative thoughts the children experienced. Unfortunately, Tuschen-Caffier et al. (2011) used a non-validated general measure of negative self-evaluation, consisting of four questions: “I can’t manage it”, “I’m excited”, “I feel insecure”, “I wonder what others watching me would think” (p. 235), which could possibly be too ambiguous as a measure of negative self-evaluation. Other studies have investigated social threat thoughts only by means of questionnaires (Rheingold et al. 2003), or focused on threat interpretations in imaginary situations (Barrett et al. 1996; Bögels and Zigterman 2000; Creswell et al. 2005; Muris et al. 2000). How social threat thoughts relate to anxiety experienced by children in real life situations could not be answered by these studies. Neither have they answered the question whether an increase in anxiety during a social evaluative task is stronger related to thoughts about social rejection (social threat thoughts) or thoughts about one’s own performance (social skills). Therefore, the first goal of this study was to investigate the relationship between social threat thoughts, perceived social skills, trait social anxiety and state anxiety during a social speech task.

If children do indeed have social threat thoughts in social situations, do they also have an increased chance of being exposed to those threats? For instance, do they have less social skills and are they therefore at risk of being rejected? Despite evidence that socially anxious children report to have given an inferior performance, it is not clear if they in fact show social skills deficits, or if their self-evaluation is biased. Some studies support the hypothesis that socially anxious children and adolescents have a social skills deficit (Beidel et al. 1999; Inderbitzen-Nolan et al. 2007), while others find that socially anxious children underestimate their social skills, while not performing worse than non-anxious children (Cartwright-Hatton et al. 2005; Morgan and Banerjee 2006). Moreover, there are no published studies that examined the link between social threat thoughts and observed social skills, even though this could give more insight into the relation between threat cognitions and actual behaviour. Thus, the second goal of this study was to investigate whether trait social anxiety, state anxiety and social threat thoughts relate to social skill deficits.

To summarize, the goals of this study were twofold. First, we investigated whether social threat thoughts and social skills perception mediate the relationship between trait social anxiety and state anxiety following a social speech task. Following the model of Hofmann (2007), we hypothesized that both more social threat thoughts and a more negative social skills perception will mediate a positive relationship between trait social anxiety and state anxiety. Our second goal was to test whether trait social anxiety is related to social skills deficits or an underestimation of social skills and if this relationship is mediated by social threat thoughts and change in state anxiety during a social speech task. Since it is unclear whether socially anxious children underestimate their skills or in fact have less social skills, we could not formulate a specific hypothesis for this research question.

## Methods

### Participants

Participants were 141 children (40 boys) aged between 8 and 13 years (*M* = 10.1, *SD* = 1.12), varying in levels of social anxiety. Children were selected from 718 children participating in a large study about anxiety and avoidance behaviour involving 11 different elementary schools. Due to time constraints, it was not possible to test all children more extensively, so a selection of 141 children was made. To increase the number of anxious children in the sample and achieve a more even distribution of social anxiety, we selected children based on the Social Anxiety Scale for Children-Revised (SASC-R; Ginsburg et al. 1998; La Greca and Stone 1993), the social anxiety subscale of the Screen for Child Anxiety Related Emotional Disorders (SCARED-71; Bodden et al. 2009) and the Behavioral Inhibition Questionnaire (BIQ; Broeren and Muris 2010), such that levels of social anxiety were more evenly distributed and approximately the same number of girls and boys scored in the lower and higher regions of self-reported anxiety. Time between screening and individual testing for the current study was approximately 3 months. To obtain an indication of the severity of the trait social anxiety of our sample, we calculated how many children scored above the clinical cut-off of 8 of the SCARED-71 social anxiety subscale. We found that 50 children (40%) scored above this clinical cut-off. The current sample partly overlapped with the samples in two studies to validate a measure of spider fear (Klein et al. in press) and a auditory interpretation task measure (Klein et al. 2016a), a study on biases in spider fear (Klein et al. in press), and a study on the specificity of interpretation biases (Klein et al. 2016b).

### Measures

#### Screen for Child Anxiety Related Emotional Disorders (SCARED-71)

The SCARED-71 measures symptoms of DSM-IV anxiety disorders, including separation anxiety, generalized anxiety disorder, panic disorder, social phobia, specific phobia, obsessive–compulsive disorder, post-traumatic stress disorder and school phobia (Bodden et al. 2009). Due to time constraints, we only used the subscale social phobia in the current study. The SCARED-71 can be used to differentiate clinically anxious from non-anxious children on a total score and on all subscales and has a good internal consistency (Bodden et al. 2009). Children score how often they experience each anxiety symptom on a 3-point Likert scale (0 = *almost never*, 1 = *sometimes*, 2 = *often*). Internal consistency of the social anxiety subscale used in the present study was good (Cronbach’s α = .88). Given that the items in the SCARED are phrased in a general or chronic way, they are considered to reflect a trait conceptualization of social anxiety (Muris et al. 2003).

#### The Children’s Automatic Thoughts Scale (CATS)

The CATS consists of 40 items that characterize different negative thoughts (e.g. “Kids are going to laugh at me”; Schniering and Rapee 2004). Social threat thoughts were measured with five items of the social threat scale of this questionnaire, supplied by Dodd et al. (2011) together with their social speech protocol: “I’m worried that I’m going to get teased; I’m going to look silly; People are thinking bad things about me; I look like an idiot; I’m afraid I will make a fool of myself”. The items are scored on a five-point scale from 0 (*not at all*) to 4 (*all the time*). The CATS has consistently shown good internal reliability, with Cronbach’s alphas ranging from .82 to .96 (Schniering and Lyneham 2007; Schniering and Rapee 2002, 2004). Cronbach’s alpha of the subset of the social threat scale in this study was .78.

#### State Anxiety

State anxiety was measured with six statements addressing different elements of anxiety. Children were asked to rate the following items on a scale from 0 to 10: anxious, excited, palpitations, funny feeling in stomach, sweating and shaking (based on: In-Albon et al. 2009). All items had two little drawings of a figure that depicted a neutral state on the left side (0) and the specific feeling on the right side (e.g. a scared expression with the item ‘anxious’; 10). Both measures of state anxiety, before and after the speech task, had good internal consistency (α = .87 and α = .88).

#### Social Speech Task

The procedure of the two minute speech task was similar to the protocol of the high anxiety condition used by Dodd et al. (2011). All children performed the task individually in a separate room at school, together with a trained research assistant. The children were told that the speech would be recorded on video and that adults would watch the videos. The children were allowed to talk about anything they wanted and the assistant gave a few examples of possible subjects. The child stood straight up, facing two cameras, one of which recorded the whole body, while the other one recorded the facial expression. The assistant sat behind the child, was not visible for the child, and did not react to the child’s utterances. When the child was silent for 10 s, the assistant gave a standardized prompt (e.g. “Could you tell something about your hobby?”). There was a maximum of three prompts, each given after 10 s of silence. The researcher wrote down how many prompts were given. The assistant ended the task if the child had not spoken after the third prompt.

### Video Ratings

#### The Performance Questionnaire

The PQ is a nine-item instrument that measures performance evaluation after a public speaking task (Cartwright-Hatton et al. 2005). There are two versions: the PQ-C for the subjective experience of the child and the PQ-O for the observer. Items are scored on a four-point scale ranging from *not very (much)* to *very (much).* Following the procedure by Miers et al. (2009), the questionnaire was split up into two scales: a social skills scale and a nervousness scale. The internal consistency for the resulting two subscales of the PQ-C in the present study was adequate (Cronbach’s α = .71 for social skills and .61 for nervousness). Two trained observers (master-students) watched the videos of the speeches and rated the participants’ performance using this questionnaire. They were blind to the anxiety level of the children and worked independently of each other. Cronbach’s alpha calculation and a regression analysis were performed to analyse the interrater agreement over a subset of 20% of the cases. Cronbach’s alpha of the nervousness scale rated by the observers was not sufficient (Cronbach’s α = .34), therefore this scale was left out of the data analysis. Cronbach’s alpha of the social skills scale was comparable with findings in previous research (α = .72). Regression analyses of the social skills scale showed that the observers did not systematically differ in their scores, and that agreement was high (*b* = .917, *t*(22) = 9.40, *p* < .001). The ratings of the first observer were a good predictor of the variance of the ratings of the other observer, *R* ^*2*^ = .794, *F*(1,24) = 88.43.

### Procedure

For all participants, parental active consent was acquired in writing prior to the study. After establishing active verbal consent from the children as well, children were individually tested in a quiet room away from the main classroom. As part of the large study, the children participated in two sessions of 1 h each. In the second session, the participants gave the 2 min impromptu speech and filled out the questionnaires. Right before the social speech task instructions, the children filled out the first state anxiety measurement. Directly after having finished the speech task, the children were asked to fill out the state anxiety measure again, as well as the social threat questions and the rating of their own performance. Trained master students supervised the sessions.

### Data Analysis

There were five children who did not fill out the social threat scale, and 12 children whose video of the social speech task was not usable for rating, so they were excluded from the data analyses. To investigate our research questions, two mediation analyses were performed with the use of the SPSS add-on PROCESS (Hayes 2013). This program is preferable over the causal steps approach of Baron and Kenny (1986), for it is a more advanced method of quantifying the intervening variable models (Hayes 2009). We used bootstrapping analysis with 5000 bootstrap resamples to generate estimates of indirect effects (Preacher and Hayes 2004). Bootstrapping is a nonparametric resampling procedure that creates an approximation of the sampling distribution of a statistic from the available data. The procedure generates point estimates and 95% confidence intervals for the indirect effects and does not require assumptions about a normal sample distribution that underlie the Sobel test. In bootstrapping analysis, the test of an indirect effect (mediation) is if the value of 0 is not between the lower and upper bound of the 95% bias corrected confidence intervals.

Bootstrapping can also be useful when data is skewed, which was the case with the scores on the social threat thoughts scale. Several children did not experience social threat thoughts at all. Since bootstrapping does not require a normal distribution, it is one of the advised methods of analysing this type of data (Delucchi and Bostrom 2004).

For the second mediation analysis, two difference scores were used. Change in state anxiety was calculated by subtracting the baseline state anxiety score from the post speech task state anxiety score. The difference between self-perception of social skills by the child and the ratings of social skills by the observers were calculated by subtracting the observer score from the child score. As a result, underestimation of social skills by the child was indicated by a negative score.

## Results

**Fig. 1:**
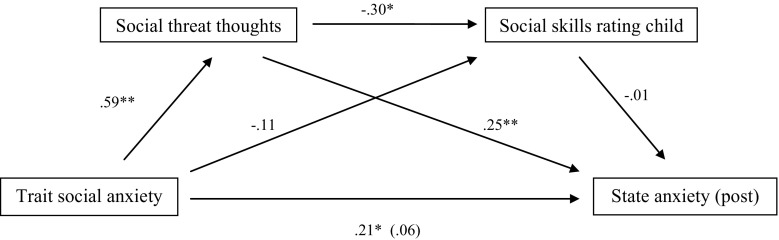
Beta values for the relationship between trait social anxiety and state anxiety as mediated by social threat thoughts and social skills rating of the child, controlled for baseline state anxiety. The beta value of the direct effect between trait anxiety and state anxiety, controlling for the mediation effect, is in *parentheses*. *p < .05; **p < .001

Table 1 presents mean scores and standard deviations for all questionnaires. First, we examined whether social threat thoughts and self-perceived social skills mediated the relationship between trait social anxiety and state anxiety. To explore this first goal, we estimated a serial multiple mediator model with these two theoretically-derived mediators (M1: social threat thoughts and M2: social skill child perception) using PROCESS (Hayes 2013). This enabled us to test the serial indirect effects of trait social anxiety on state anxiety scores via social threat thoughts and social skills perception (see Fig. 1). Baseline state anxiety score was entered as a covariate. Results indicated that the total effect of trait social anxiety on post-state anxiety scores was significant (β = 0.21, *t* = 4.11, *SE* = .05, *p* < .001). As expected, we found that the indirect effect of trait social anxiety on state anxiety via social threat thoughts was statistically different from zero (β = .24, 95% CI = .10 to .40). The indirect effect of trait social anxiety on state anxiety via self-perceived social skills was not significant (β = .001, 95% CI = −.02 to .05). Furthermore, the predicted model of the serial indirect effect of trait social anxiety on state anxiety via social threat thoughts and self-perceived social skills was not supported, as the indirect effect was not statistically different from zero (β = .003, 95% CI = −.02 to .04). This means that social threat thoughts mediated the relationship between trait social anxiety and state anxiety, as well as the relationship between trait social anxiety and self-perceived social skills, even while controlling for baseline state anxiety. Contrary to our expectations, negative self-perceived social skills did not relate to a higher state anxiety after the task.

**Table 1:** Means and standard deviations

Scale	M	SD
SCARED-soc (sumscore)	6.76	4.96
Social threat thoughts	1.14	1.63
SA baseline	0.87	1.15
SA post	1.51	1.80
Δ SA	0.63	1.07
PQ-C skills	2.21	0.52
PQ-O skills	2.33	0.50
Δ PQ skills	−.12	0.79

SCARED-soc = trait social anxiety; SA = state anxiety; PQ-C skills = social skills child perception; PQ-O skills = social skills observer perception

Our second research goal was to test whether trait social anxiety is related to social deficits or an underestimation of skills and if this relationship is mediated by social threat thoughts and change in state anxiety on the social speech task. First, to investigate if trait anxiety is related to social skill deficits or an underestimation of skills, we calculated correlations to investigate whether children with higher trait social anxiety were rated lower by the observers on their social skills than low trait anxious children, or if these children underestimated their skills. As is shown in Table 2, none of the anxiety-related measures correlated with the rating of social skills by the observer (trait social anxiety: *r* = .03, *p* = .76). However, trait social anxiety did correlate significantly with the difference score of the social skills rating (child rating minus observer rating), showing a negative relationship (*r* = −.18, *p* = .042), which indicates that children with higher trait anxiety rated their social skills lower than the observers. When looking at the slope of this relationship (*y* = 0.08−0.03*x), we found that children with a trait social anxiety score of 2.67 score or lower, rated themselves higher or equal to the observers. When trait social anxiety was higher than 2.67, children underestimated their social skills compared to observers. Since the clinical cut-off for this subscale is 8, we found that children scoring under this cut-off score also had the tendency to slightly underestimate their social skills compared to the observers. These results indicate that trait socially anxious children do not demonstrate a social skills deficit, but underestimate their social skills.

**Table 2:** Bivariate correlations between measures of cognitions, affect and behaviour

	SCARED-soc	Social threat thoughts	SA baseline	SA post	Δ SA	PQ-C skills	PQ-O skills
Social threat thoughts	.63**						
SA baseline	.34**	.31**					
SA post	.47**	.52**	.82**				
Δ SA	.39**	.51**	.24*	.74**			
PQ-C skills	−.25*	−.33**	.01	−.10	−.16		
PQ-O skills	.03	−.08	.03	−.01	−.06	−.21*	
Δ PQ skills	−.18*	−.17	−.02	−.06	−.07	.79*	−.76**

SCARED-soc = trait social anxiety; SA = state anxiety; PQ-C = skills social skills child perception; PQ-O skills = social skills observer perception

* *p* < .05; ** *p* < .001

**Fig. 2:**
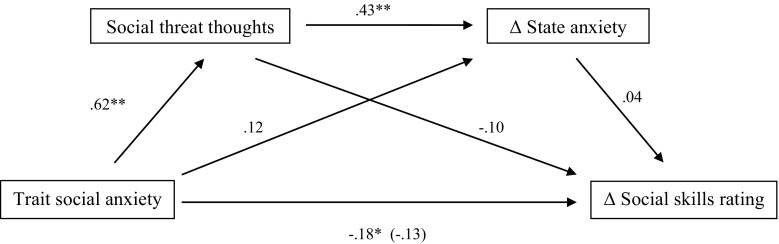
Beta values for the relationship between trait social anxiety and difference score of social skills rating as mediated by social threat thoughts and change in state anxiety. The beta value of the direct effect between trait anxiety and the difference score of social skills rating, controlling for the mediation effect, is in *parentheses*. *p < .05; **p < .001

To further examine whether the relationship between trait social anxiety and social skills underestimation is mediated by social threat thoughts and change in state anxiety during the speech task, we performed a serial multiple mediation analysis with two mediators (M1: social threat thoughts and M2: change in state anxiety; see Fig. 2). Results showed that the total effect of trait social anxiety on the social skills difference score was significant (β = −0.18, *t* = −2.06, *SE* = .09, *p* = .042). The indirect effect of trait social anxiety on social skills difference score via social threat thoughts was not statistically different from zero (β = .07, 95% CI = −0.21 to 0.60). Also, the indirect effect of trait social anxiety on social skills difference score via state anxiety change score was not statistically different from zero (β = .004, 95% CI = −0.02 to 0.05). Finally, the predicted model of the serial indirect effect of trait social anxiety on social skills difference score via social threat thoughts and state anxiety change score was not supported, as the indirect effect was not statistically different from zero (β = .01, 95% CI = −0.05 to 0.7). These results demonstrate a relationship between trait social anxiety and social skills underestimation: the higher the trait social anxiety, the more children underestimated their social skills. This effect was not mediated by social threat thoughts or change in state anxiety.

## Discussion

The first goal of this study was to investigate whether social threat thoughts and social skills perception of the child mediated the relationship between trait social anxiety and state anxiety after a social speech. We found that social threat thoughts were related to lower self-perception of social skills. Furthermore, we found that social threat thoughts mediated the relationship between trait social anxiety and state anxiety after a speech task, even when controlling for baseline state anxiety. Self-perception of social skills did not mediate the relationship between trait and state anxiety. It is important however, to keep in mind that the two mediators were examined at the same time-point, meaning that it is not possible to derive any causal hypotheses from this model. The second goal was to test whether trait social anxiety is related to a social skills deficit or to an underestimation of social skills, and if this relationship is mediated by social threat thoughts and change in state anxiety on the social speech task. We found that children with higher trait social anxiety tended to underestimate their performance, but they were not observed to have poorer social skills than children with lower trait social anxiety. Moreover, the relationship between trait social anxiety and underestimation of skills was not mediated by social threat thoughts or change in state anxiety.

The present study provides evidence that two important aspects of the most influential social anxiety theories are applicable to children (Clark and Wells 1995; Hofmann 2007; Rapee and Heimberg 1997). First, the current results support that social threat thoughts are contributing to the prediction of state anxiety of children in social evaluative situations. Second, social skills perception did not relate to change in state anxiety, which indicates that social threat thoughts are more relevant in the process of elevated state anxiety in trait socially anxious children than social skills perception. However, we did find that these two cognitive factors had a significant negative correlation; children who displayed more social threat thoughts perceived their social skills as being lower. This result is in line with other studies that show that socially anxious children have a specific negatively biased cognitive processing style (Ferreri et al. 2011) and is an addition to the already existing child literature about the importance of threat-based interpretations and threat responses as maintaining factor in anxiety disorders (Manassis 2013). This finding also corresponds with adult studies that found that individuals with a social anxiety disorder form negative mental representations, which is based on how they think potential evaluators would view them (Hackmann et al. 1998; Wells et al. 1998). It would be worthwhile for further research to explore if the presence of social threat thoughts is a prognostic risk factor or maintaining factor for the development of social anxiety.

With regard to the second research question, we did not find evidence for a social skill deficit; we only found a non-mediated relationship of trait social anxiety with an underestimation of social skills. We found that children generally tended to underestimate their social skills compared to observers, and this underestimation increased with a higher trait social anxiety. These findings are consistent with cognitive theories of social anxiety, which assume that socially anxious people have the tendency to underestimate their skills, and overestimate risk of rejection (Clark 2001; Hofmann 2007).

Some limitations of the current study should be mentioned. First, we exposed the children to a video camera and an experimenter, which is not representative of usual social situations. To increase the ecological validity of these findings, it may be better to test the children in a more realistic setting, such as in a classroom or during interaction with peers (Spence et al. 1999), even though it should be noted that there might be more interfering factors in a realistic setting that cannot be easily measured and controlled. Second, we only measured social threat thoughts after the social speech task. It would be useful to measure these thoughts at more time points. Third, we only included children with varying levels of social anxiety, and the results might differ for children with a diagnosed social anxiety disorder. It is possible that children with a social anxiety disorder are even more vigilant for threat and therefore their social threat thoughts might be triggered by milder threats (Mogg and Bradley 1998). Future studies should include a clinically anxious group, before firm conclusions can be drawn. Furthermore, our study focussed on social anxiety only and it would be worthwhile to investigate if social treat thoughts are specific to social anxiety or if they arise in a variety of anxiety disorders. Finally, to gain more insight in the relationship between social threat thoughts and the maintenance of social anxiety, it would be relevant to measure the relation between social threat thoughts and behaviour. For instance, does the presence of social threat thoughts predict automatic approach–avoidance behaviour (Heuer et al. 2007). If so, this would be in line with cognitive theories of social anxiety that state that social threat interpretations lead to avoidance behaviour (Hofmann 2007). Some evidence for this hypothesis has been found in adults by Rachman et al. (2000), who reported that post-event rumination was related to anxiety during a social situation and avoidance of comparable situations in the future.

### Clinical Relevance

This study showed that an increase in anxiety during social situations could be partly explained by the presence of cognitions about being rejected by others. Challenging these social threat thoughts in children might be an important component in treatment, as evidence suggests that change of threat expectancies regarding the likelihood of aversive events could be the working mechanism in anxiety treatments, as opposed to habituation (Craske et al. 2014). Thus, exposure to social situations without taking the social threat expectancies in account might even be counter productive. Anxious children will continue to feel rejected, even though there is no objective evidence that they are performing worse than other children. Since there are already treatment protocols for adults that focus on these expectancies and include (video) feedback to change these social threat thoughts (Hofmann and Otto, 2008), it is very relevant to study if this approach would also be beneficial for children.
